# Patients’ attitudes to disease prevention in inflammatory bowel disease: a US-based survey

**DOI:** 10.1093/crocol/otag004

**Published:** 2026-02-03

**Authors:** Mary Harkins-Schwarz, Catarina Bravo, Manasi Agrawal, Jean Frederic Colombel, Ryan Ungaro, Laura Wingate, Joana Torres, Alan Moss

**Affiliations:** Crohn’s & Colitis Foundation, New York, NY, United States; Division of Gastroenterology, Hospital Beatriz Ângelo, Loures, Portugal; Dr Henry D. Janowitz Division of Gastroenterology, Icahn School of Medicine at Mount Sinai, New York, NY, United States; Center for Molecular Prediction of Inflammatory Bowel Disease (PREDICT), Department of Clinical Medicine, Aalborg University, Copenhagen, Denmark; Department of Environmental Medicine, Icahn School of Medicine at Mount Sinai, New York, NY, United States; Dr Henry D. Janowitz Division of Gastroenterology, Icahn School of Medicine at Mount Sinai, New York, NY, United States; Division of Gastroenterology, Icahn School of Medicine at Mount Sinai, New York, NY, United States; Crohn’s & Colitis Foundation, New York, NY, United States; Division of Gastroenterology, Hospital Beatriz Ângelo, Loures, Portugal; Division of Gastroenterology, Hospital da Luz, Lisbon, Portugal; Faculdade de Medicina, Universidade de Lisboa, Lisbon, Portugal; Crohn’s & Colitis Foundation, New York, NY, United States

**Keywords:** inflammatory bowel disease (IBD), prevention, screening, diagnosis, treatment, risk, benefit, first-degree relative (FDR)

## Abstract

**Background:**

Recent advances in biomarkers have identified at-risk cohorts for inflammatory bowel disease (IBD), and potential interception strategies to prevent disease onset are in progress. We sought to understand patient and family members’ views on IBD prevention, as they are key stakeholders in future adoption of prevention recommendations.

**Methods:**

A workgroup of patient advocacy organizations and researchers adapted a survey for completion by the IBD community residing in the United States. All responses were anonymous. Descriptive results, and comparisons, were undertaken of pooled responses.

**Results:**

One thousand five hundred forty-five respondents completed the survey. Most respondents (93%, *n = *1,421) would be interested in taking a test to predict their or their family’s risk of developing IBD in the future. Almost all respondents were interested in taking preventative treatment to prevent IBD; 40% expressed an unconditional interest in the treatment, but 59% reported it would be dependent on the risks and effectiveness of the treatment. Lifestyle measures were the most preferred option to prevent IBD. There was no significant difference in proportion of patients who were willing to take a test or prevention treatment based on relationship to IBD (have IBD, first-degree relative of someone who has IBD, or parent of someone with IBD).

**Conclusions:**

Most people affected by IBD in the United States agree with taking proactive measures to prevent IBD. A lifestyle intervention (diet, exercise) is favored over a pharmaceutical approach by these respondents. Relationship to IBD did not influence the magnitude of the agreement.

## Graphical abstract

**Graphical Abstract:**
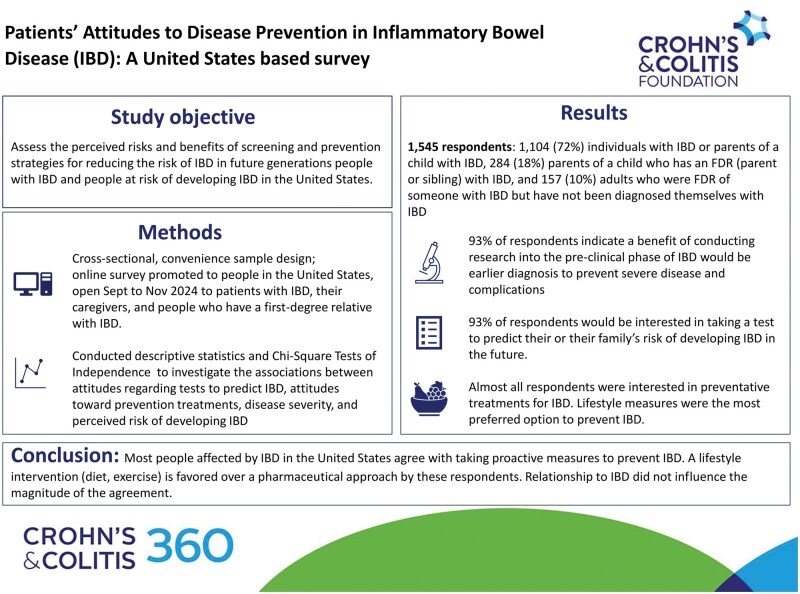

## Introduction

Inflammatory Bowel Disease (IBD) is a chronic condition of intestinal inflammation, which affects an estimated 2.39 million Americans, including more than 100,000 children.^1^^,^^2^ There is significant morbidity for people with IBD, including surgeries, hospitalizations, growth failure and impaired productivity associated with lifelong IBD.^3^^,^^4^ In addition, people with IBD typically experience high health care costs, negative impact on work productivity and quality of life.^5–9^ There is currently no cure for IBD. The historical approach to treating IBD has focused on diagnosis, and then inducing remission in patients with active disease.^10^^,^^11^ A delay in diagnosis is common, with an average time from symptom-onset to diagnosis of 4-8 months.^12^ Delayed diagnosis is linked to higher risk of developing severe complications, including the need for surgery.^12^^,^^13^ Earlier diagnosis of IBD is critical for initiating interventions and care, preventing disease progression, and enabling better patient outcomes.

Recent research has focused on advancing the science of predicting and preventing IBD.^14^ Researchers examined fecal, genetic, and intestinal makers of the pre-clinical phase among first degree relatives (FDR) of those with Crohn’s disease and identified biomarkers that can precede the diagnosis of IBD by years.^15–18^ In parallel, studies of serum markers in healthy military personnel have reported biomarkers associated with disease risk that are detectable up to 7 years before a subsequent clinical diagnosis of IBD.^19^ The convergence of these data has supported recent trials aimed at interventions to delay Crohn’s disease in at-risk individuals.^20–22^

While science has progressed our understanding of potential screening approaches, the attitude of the IBD patient community to these concepts remains unknown. A recent survey of parents of children at-risk for IBD development (based on family history) and first-degree relatives of people with IBD in Europe reported high levels of acceptance of screening and lifestyle interventions to prevent IBD.^23^ However, attitudes to health prevention approaches to rare diseases may differ in the United States, where system-wide economic barriers to medical care exist at the individual level.^24^ In this study, we sought to assess the perceived risks and benefits of screening and prevention strategies for reducing the risk of IBD in future generations among a US population, including people with IBD and people at risk of developing IBD. We also examined the relationship between disease severity and perceived likelihood of developing IBD with willingness to take a screening test or a preventive treatment.

## Materials and methods

### Design

The survey was adapted from one originally developed and administered to a European cohort, focused on first-degree relatives.^23^ An advisory panel comprised of representatives from patient advocacy organizations [names redacted for review], researchers, and healthcare professionals reviewed and revised the survey to clarify questions, reduce survey burden, and tailor questions for patients receiving care primarily in a United States health care system.

The survey (Supplementary Data) consisted of 40 questions structured across six parts: characteristics of person with IBD (respondent or relative with IBD) (e.g., type of disease, age diagnosed, impact of disease (7 questions)); impact of IBD on quality of life (1 question); knowledge of IBD (6 questions); attitudes about predicting IBD (4 questions); perceptions about clinical research to understand pre-clinical phase of IBD (1 question); perceptions about preventive treatment for IBD (8 questions); and perceived risk of developing IBD (1 question). Finally, participants were provided an open-ended text box to share their thoughts about predictive tests and preventive treatments/interventions. Survey respondent demographic characteristics and familial relationship to IBD (e.g., patient, sibling has IBD, child has IBD) were also collected.

### Participants

The survey was open to adults with IBD; adults who had a first-degree relative with the disease (parent, sibling, child with IBD); and adults who have a child at-risk for IBD development (either because the parents had IBD themselves or another child living with IBD). The following criteria were used to determine respondent’s survey eligibility and inclusion in the final dataset: (1) have IBD or relationship with an individual with IBD; (2) age ≥18 years; (3) completion of >80% of questions, excluding demographic characteristic questions), and (4) completed response to question regarding familial relationship to IBD. For the questions regarding risks and benefits of screening and treatment, respondents already diagnosed with IBD were instructed to answer questions in the context of their at-risk family members, and their disease experience.

### Procedure

The survey was conducted via an online survey platform (SurveyMonkey; San Mateo, CA, United States) and fielded from September through November 2024 (8 weeks). The survey was only available in English and open to anyone who accessed the survey link (convenience sampling approach). The lead organization [name redacted for review] disseminated recruitment communications through social media and constituent listservs (including patients, caregivers, and healthcare professionals). Partner networks [names redacted for review] were provided a survey link and asked to share with their constituents. All responses were collected anonymously, and no incentive was offered.

### Ethical considerations

The study was reviewed by the NorthStar Review Board and considered exempt from IRB oversight under 45 CFR 46 (the Common Rule) §104(d)(2)(i).

### Statistical analysis

Statistical analyses were performed using SPSS (IBM Corp. Released 2024. IBM SPSS Statistics for Windows, Version 30.0.0.0 Armonk, NY: IBM Corp) statistical software. This paper presents the findings from our analysis. Descriptive statistics were used to describe demographic characteristics and responses regarding attitudes toward prediction and prevention of IBD. For the types of tests and types of interventions that respondents ranked in order of willingness to take, we reverse scored each item (9 = most willing to take and 1 = least willing to take) and calculated the mean for each item.

To investigate the associations between attitudes regarding tests to predict IBD, attitudes toward prevention treatments, disease severity, and perceived risk of developing IBD, we conducted Chi-Square Tests of Independence for each pair of variables. Specifically, we examined the relationships between attitudes regarding tests to predict IBD and disease severity; prevention treatments and disease severity; tests to predict IBD and perceived risk of developing IBD; and prevention treatments and perceived risk of developing IBD. *P* values of <.05 were considered to be statistically significant.

## Results

### Population

**Figure 1:**
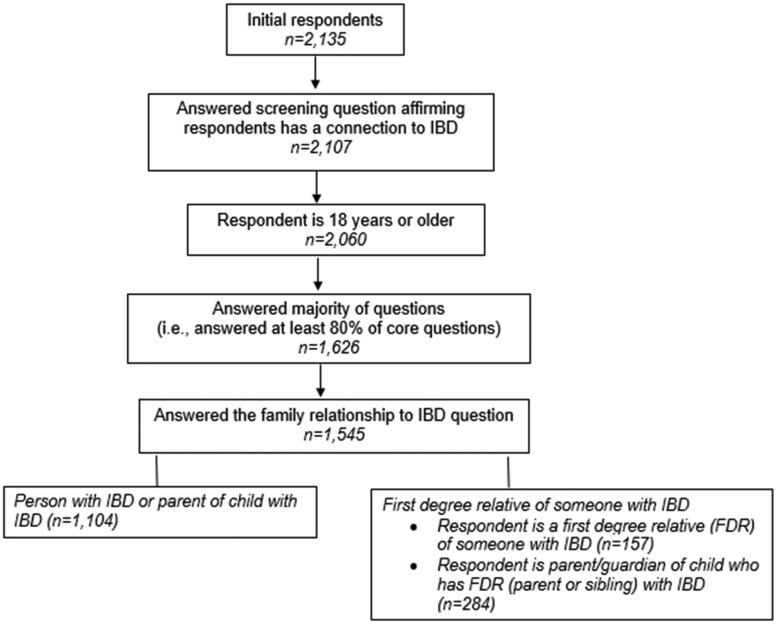
Flow diagram depicting survey eligibility.

The initial responses included 2,135 answers. Of these, 590 responses were ineligible (e.g., incomplete survey, under the age of 18; or lacked a connection to IBD). The remaining 1,545 respondents were coded as *complete* and used for analysis (Figure 1). Of the 1,545 respondents, 1,104 (72%) were individuals with IBD or parents of a child with IBD, 284 (18%) were parents of a child who has an FDR (parent or sibling) with IBD, and 157 (10%) were adults who were FDR of someone with IBD but have not been diagnosed themselves with IBD (Figure 1). Most respondents were 26 to 64 years old (73%, *n *= 1,116), identified as female (80%, *n *= 1,226), and had a college degree or higher: 38% had a bachelor’s degree (*n *= 581) and 36% had a Master’s degree (*n *= 555) (Table 1**)**. All 50 states and the District of Columbia were represented, except for North Dakota. Nine percent of respondents (*n *= 138) did not identify a state.

**Table 1:** Demographic characteristics of respondents.

	Total (*N *= 1,545)		Parent of a child at risk of IBD (*n *= 284)		Adult at risk of IBD (FDR) (*n *= 157)		Patient with IBD or parent of child with IBD (*n *= 1,104)	
	%	*#*	%	*#*	%	*#*	%	*#*
**Age**								
** 18-25 years**	11%	164	5%	14	26%	40	10%	110
** 26-64 years**	73%	1,116	83%	233	71%	110	71%	773
** 65 years and older**	16%	240	12%	35	4%	6	18%	199
**Gender**								
** Female**	80%	1,226	85%	240	75%	118	79%	868
** Male**	19%	288	14%	40	23%	36	19%	212
**Non-binary, other, prefer not to answer**	1%	24	1%	3	2%	3	2%	18
**Race**								
** White, non-Hispanic**	82%	1250	89%	248	77%	119	81%	883
** Black, non-Hispanic**	5%	73	2%	6	7%	11	5%	56
** Asian, non-Hispanic**	5%	72	3%	8	3%	5	5%	59
** Other, non-Hispanic**	0.7%	10	1%	3	0%	0	1%	7
** Multiracial, non-Hispanic**	3%	39	2%	5	3%	4	3%	30
**Ethnicity**								
** Hispanic**	5%	83	4%	10	11%	16	6%	57
**Education**								
** Less than high school**	1%	10	0%	1	1%	2	1%	7
** High school or GED**	6%	88	3%	8	10%	15	6%	65
** Some college/associates degree**	17%	261	20%	31	18%	192	17%	261
** Bachelor’s degree**	38%	581	34%	107	38%	60	38%	414
** Master’s degree or higher**	36%	555	44%	123	27%	42	35%	390
** Other/prefer not to disclose**	3%	38	2%	5	5%	7	2%	26

### Disease characteristics

Most respondents (or their family member affected with IBD) had Crohn’s disease (67%, *n *= 1,028) and were diagnosed on average at 25.4 years of age. Most respondents reported moderate to severe disease (85%, *n *= 1,307) as defined as having received biologics/biosimilars, targeted synthetic small molecules, and/or surgery for their IBD (Table 2).

**Table 2:** Disease characteristics of respondent or person affected with IBD.

	Total (*N *= 1,545)		Parent of a child at risk of IBD (*n *= 284)		Adult at risk of IBD (FDR) (*n *= 157)		Patient with IBD or parent of child with IBD (*n *= 1,104)	
	Mean ± SD		Mean ± SD		Mean ± SD		Mean ± SD	
	%	*#*	%	*#*	%	*#*	%	*#*
**Age of diagnosis**	25.4 ± (14.7)		23.7 ± (13.3)		25.2 ± (13.2)		25.8 ± (15.2)	
**Type of IBD**								
** Crohn’s disease**	67%	1028	70%	199	70%	110	65%	719
** Ulcerative colitis**	30%	468	27%	76	29%	45	31%	347
** Indeterminate colitis or unknown**	3%	49	3%	9	1%	2	4%	38
**Disease severity**								
** Moderate to severe disease**	85%	1307	88%	249	84%	130	84%	928
** Mild disease**	15%	232	12%	35	16%	24	16%	173

### Perceived benefits of conducting research into pre-clinical phase of IBD

Over 90 percent of respondents (93%, *n *= 1,431) indicated a perceived benefit of conducting research into the pre-clinical phase of IBD would be earlier diagnosis to prevent severe disease and complications. Most respondents also indicated that improving knowledge of IBD in general (83%, *n *= 1,285); predicting the risk of IBD developing (82%, *n *= 1,267); preventing the disease from developing (79%, *n *= 1,218); and development of new therapies (79%, *n* = 1,214) could be the benefits of conducting clinical research into the pre-clinical phase of IBD.

### Attitudes toward predicting IBD

Most respondents (93%, *n = *1,421) indicated that if there was a test that could predict their risk or their family’s risk of developing IBD in the next few years, they would like to take it. Among these respondents, 77% (*n *= 1,090) reported that the accuracy of the test would influence if they would take it, while 23% (*n *= 331) reported that they would take the test regardless of accuracy (Table 3). There was a significant relationship between attitudes toward predicting IBD and disease severity, *X*^2^(1, N = 1,537) = 8.36, *p* = .004. Respondents with mild disease were more likely to report that they would not or were not sure that they would take a test to predict their risk of developing IBD compared to respondents with moderate to severe disease. Regardless of whether respondents perceived a medium to low likelihood or high likelihood of developing IBD, respondents answered similarly regarding attitudes toward taking a test to predict IBD. There was not a significant relationship between attitude toward taking a test to predict IBD and perceived likelihood of developing IBD, *X*^2^*(1, N *= 1,517) = 1.41, *p* = .235.

**Table 3:** Attitudes toward prediction and prevention of IBD.

	Total (*N *= 1,543)		Parent of a child at risk of IBD (*n *= 284)		Adult at risk of IBD (FDR) (*n *= 157)		Patient with IBD or parent of child with IBD (*n *= 1,104)	
	%	*#*	%	*#*	%	*#*	%	*#*
**Willingness to take a test, if it existed, that could predict their or their family’s risk of developing IBD in the next few years**								
**Yes, but it would depend on accuracy of the test**	71%	1,090	75%	212	67%	105	70%	773
**Yes, regardless of test accuracy**	22%	331	17%	47	25%	39	22%	245
**I am not sure**	6%	92	6%	17	6%	10	6%	65
**No, I would rather not know**	2%	30	3%	8	1%	2	2%	20
**Willingness to take a preventive treatment, if it existed, capable of reducing the risk of they or their family member developing the disease**								
**Yes**	40%	612	35%	100	34%	53	42%	459
**No**	1%	11	1%	3	3%	4	0%	4
**Depend on effectiveness, difficulty, and risks of treatment/intervention**	60%	918	64%	180	64%	100	60%	638
**Level of effectiveness needed to accept preventive treatment**								
**100% risk reduction (completely get rid of developing the disease)**	6%	99	4%	11	6%	9	7%	79
**80% risk reduction (8 out of 10 people stop developing the disease)**	23%	360	20%	57	31%	48	23%	255
**Risk reduction 50% (5 out of 10 people stop developing the disease)**	9%	143	12%	35	6%	9	9%	99
**It depends on the type of treatment/intervention**	34%	528	42%	119	34%	53	32%	356
**Even if the risk of developing the disease remained the same, decreasing the impact of the disease or delaying its development would already be worth it**	27%	412	22%	62	24%	38	28%	312
**Acceptable level of risk for minor adverse effects of a treatment to prevent IBD**								
**Fewer than 1 in 10 people experience minor adverse effects**	25%	383	21%	60	23%	35	26%	288
**Up to 1 in 10 people experience minor adverse effects**	18%	270	17%	48	28%	43	16%	179
**Up to 2 in 10 people experience minor adverse effects**	17%	254	17%	49	17%	26	16%	179
**Up to 4 in 10 people experience minor adverse effects**	8%	129	9%	26	7%	11	8%	92
**Would accept a higher risk (out of 10 people, 6 or more experience minor adverse effects) if the treatment were 100% effective in preventing the disease**	33%	498	35%	99	26%	40	33%	359
**Acceptable level of risk for major adverse effects of a treatment to prevent IBD**								
**Up to 1 in 100 people experience major adverse effects**	58%	884	62%	173	56%	88	57%	623
**Up to 5 in 100 people experience major adverse effects**	20%	304	17%	48	23%	35	20%	221
**Up to 10 in 100 people experience major adverse effects**	7%	105	5%	14	10%	15	7%	76
**Would accept a higher risk (out of 100 people, 11 or more experience major adverse effects) if the treatment were 100% effective in preventing the disease**	15%	230	16%	46	10%	15	16%	169
**Acceptable length of time would take a preventive pharmacological treatment (medicine)**								
**8 weeks or less**	14%	206	13%	35	9%	14	14%	157
**9 weeks to 6 months**	13%	202	13%	36	16%	25	13%	141
**7 months to 1 year**	11%	160	12%	34	14%	21	10%	105
**More than 1 year to 4 years**	7%	107	7%	20	8%	12	7%	75
**5-10 years**	2%	29	1%	3	1%	1	2%	25
**Would accept a treatment for life, if it was 100% effective in preventing the disease**	54%	825	54%	153	53%	83	54%	589
**Acceptable length of time would take/participate in a preventive non-pharmacological treatment (physical exercise, diet, etc.)**								
**8 weeks or less**	4%	64	4%	10	5%	8	4%	46
**9 weeks to 6 months**	5%	73	5%	13	5%	8	5%	52
**7 months to 1 year**	6%	91	5%	15	7%	11	6%	65
**More than 1 year to 4 years**	5%	80	4%	12	10%	15	5%	53
**5-10 years**	3%	46	3%	8	4%	6	3%	32
**Would accept a treatment for life, if it was 100% effective in preventing the disease**	77%	1,183	79%	224	69%	108	77%	851

Most respondents (86%, *n *= 1,330) reported that the advantage of taking a test to predict IBD risk would be to provide the opportunity to diagnose and begin treatment as early as possible. Most respondents (79%, *n *= 1,213) also reported that a test would allow them the opportunity to change their lifestyle (e.g., diet, exercise), take medication, or have surgery to reduce the risk of developing IBD. More than half of respondents (57%, *n *= 877) reported that a test would allow them the opportunity to explain the disease to family members in a timely manner or learn more about what IBD is and what it is like to live with the disease. Finally, one-third of respondents (34%, *n *= 527) reported that a test would provide them with an opportunity for activities or trips which may be more difficult after disease onset.

**Figure 2:**
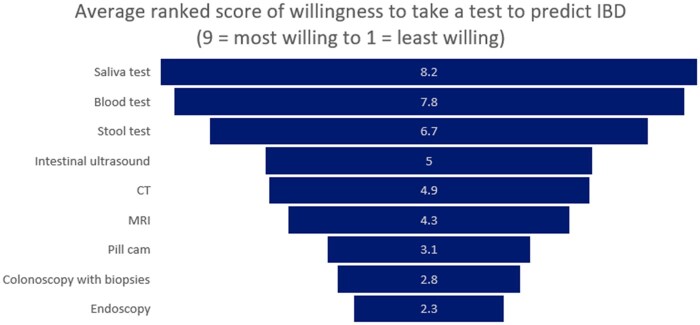
Tests to predict IBD ranked by willingness to take on a scale from 9 = most willing to take to 1 = least willing to take.

Seven out of ten respondents (73%, *n *= 1,121) reported that a disadvantage to taking a test to predict if they or a family member will develop IBD is the risk of inaccurate results. Additionally, 71% of respondents (*n *= 1,097) reported that it may generate anxiety if they have a high risk for the disease and anticipate the disease developing. Finally, six out of ten respondents (61%, *n *= 937) reported a disadvantage of taking a test is worrying about the risk of developing the disease in the absence of an intervention. Regarding willingness to take different types of tests to predict IBD, respondents ranked a saliva test highest (average score of 8.2 on scale from 9—most willing to take to 1 - least willing to take), followed by blood test (7.8), and then a stool test (6.7) (Figure 2).

**Figure 3:**
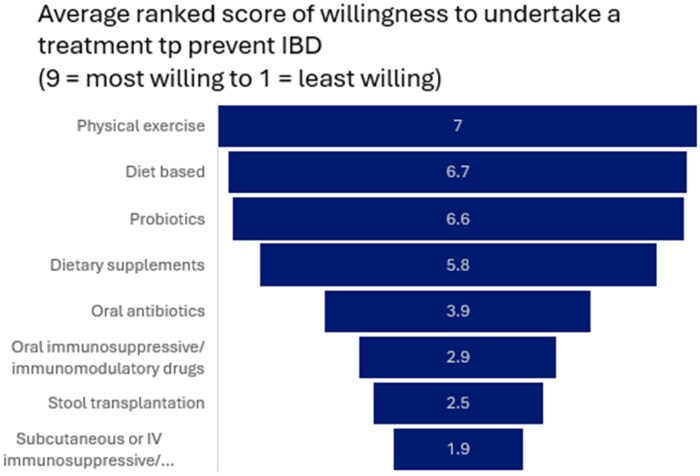
Treatment to reduce risk of developing IBD ranked by willingness to take on a scale from 9 = most willing to take to 1 = least willing to take.

### Perceptions about preventive treatments

Two out of five respondents (40%, *n *= 612) reported that if there were some kind of preventive treatment capable of reducing the risk of them or their family member developing IBD, they would like to try it; 59% of respondents (*n *= 918) indicated that their willingness would depend on the effectiveness, difficulty, and risks of the treatment/interventions. One percent (*n *= 11) of respondents indicated they would not be willing to take preventive treatment. There was no relationship between attitude toward taking a preventive treatment to reduce their risk of developing IBD (“yes” compared to “depends on”) and disease severity, *X*^2^*(1, N *= 1,524) = 0.00, *p* = .987. There was a significant relationship between attitude toward taking a preventive treatment to reduce IBD and perceived likelihood of developing IBD, *X*^2^*(1, N *= 1,505) = 9.06, *p* = .003: respondents who perceived their likelihood of developing IBD as low to medium were more likely than those who perceived their likelihood as high to report that their willingness to take a preventive treatment would depend on the effectiveness, difficulty, and risks of the treatment (Figure  3).

Most respondents (94%, *n *= 1,450) reported that in order for them or their family to accept doing it, a preventive intervention should reduce or eliminate the risk of the disease developing; 71% (*n *= 1,088) of respondents reported a goal should be to make the disease less severe, and 55% (*n *= 848) reported that a goal should be to delay the onset of disease.

Regarding willingness to take a treatment to reduce the risk of developing IBD, respondents ranked physical exercise the highest (average score of 7 on a scale from 9—most willing to take to 1 - least willing to take), followed by diet based (6.7), probiotics (6.6), and dietary supplements (5.8). Subcutaneous or IV immunosuppressive/immunomodulatory drugs were ranked last (average score 1.9) (Figure 2).

Respondents had mixed views on how effective a preventive treatment or strategy would have to be for them or their family to accept it. Six percent of respondents (*n *= 99) reported they would accept a treatment only if it had 100% risk reduction (Table 3). Over twenty percent of respondents (23%, *n *= 360) would accept an 80% risk reduction. Approximately one out of four respondents (27%, *n *= 412) reported they would accept treatment even if the risk of developing the disease remained the same because decreasing the impact of the disease or delaying the development would already be worth it.

Respondents’ willingness to accept minor and major adverse effects of preventive treatments decreased as the level of risk of the side effects increased. One out of four respondents (25%, *n *= 383) would use a treatment to prevent IBD from occurring if the risk of minor adverse effects (e.g., headache, nausea or vomiting) were fewer than 1 in 10 people (Table 3). A third of respondents (33%, *n *= 498) would accept a high risk (e.g., out of 10 people 6 or more experience minor adverse effects) if the treatment were 100% effective in preventing the disease.

More than half of respondents (58%, n = 884) would accept serious adverse effects (e.g., hospitalizations, invasive tests, intravenous treatments) of a treatment to prevent IBD if the risk of the effects was 1 in 100 people (Table 3). Fifteen percent of respondents (*n *= 230) reported that they would accept a higher risk (e.g., out of 100 people, 11 or more experience major adverse effects) if the treatment were 100% effective in preventing the disease.

Respondents’ willingness to accept pharmacological treatment decreased as the expected length of treatment increased. Fourteen percent of respondents (*n *= 206) would accept pharmacological treatment if the duration of treatment was eight weeks or less (Table 3). Only two percent of respondents (*n *= 29) would accept pharmacological treatment if the duration was five to ten years. However, more than half of respondents (54%, *n *= 825) would accept a treatment for life if it was 100% effective in preventing the disease. In terms of non-pharmacological treatments, most respondents (77%, *n *= 1,183) would accept a non-pharmacological treatment (e.g., physical exercise, diet) for life if it were 100% effective in preventing the disease.

## Discussion

The concept of prevention of IBD is a relatively novel one for the IBD community, as research, education and advocacy efforts have focused on patients with established disease to date. The advent of several research projects and publications on preclinical IBD, and serum biomarkers of risk, have provided an opportunity to identify at-risk groups who may benefit from monitoring to reduce time to diagnosis and interventions to delay or prevent disease.^15^^,^^19^ Other autoimmune conditions, such as type 1 diabetes, have revealed well-characterized high risk pre-clinical groups, and an FDA-approved therapy now exists to delay disease onset in these individuals.^25^^,^^26^ In addition, researchers are beginning to explore how communicating risks for rheumatoid arthritis using genetics, biomarkers and lifestyle factors may motivate FDR to adopt lifestyle changes to reduce their risk of developing the disease.^27^ These data have provided the impetus for the Crohn’s & Colitis Foundation and other patient-advocacy organizations to include a greater understanding of preclinical pathways and mechanisms in their research strategy and a vision for preventing IBD in their strategic plan.^28^^,^^29^

A key component of any pre-clinical screening and interception discussions is willingness of at-risk individuals and their caregivers/parents to undergo screening, and healthcare systems to pay for such testing.^30–33^ An informed, participatory public is the cornerstone of any public health intervention.^34^ In parallel, research funders and government agencies are informed by public acceptance and advocacy for specific approaches. In this setting, as awareness that IBD has a preclinical phase which may be amendable to early intervention, an understanding of patient and caregiver willingness to undertake screening and prevention interventions is critical for success.

This study shows that there is overwhelming interest among people with IBD and their family members for tests to predict risk of developing IBD and prevention treatments to reduce risk of developing IBD, reducing its severity, and delaying onset. While minimizing the risk of developing inflammatory bowel disease should be the primary objective of any treatment, it is equally important to focus on reducing the severity and delaying the onset of the disease to enhance patient outcomes and reduce societal healthcare costs. People with IBD and first-degree relatives, rightfully so, express concern about accuracy of any tests and anxiety related to learning about disease risk. Learning about disease risk may also negatively impact mental health and quality of life. In addition, a false positive test may result in unwarranted anxiety as well as potentially leading to unnecessary treatments and interventions.

The findings from this study echo results from the recent study by Bravo et al. which found that among a population of primarily parents of children with IBD and first-degree relatives in Europe, respondents prefer lifestyle interventions, such as diet, physical exercise, and probiotics compared to pharmacological interventions. Generally, lifestyle interventions are considered safer than pharmacological interventions. Compared to medications, lifestyle interventions, such as changing diet or exercise routine, have minimal side effects, are less likely to result complications, and are often less costly. In addition, lifestyle interventions can be tailored to patient preferences and belief systems. Conversely, lifestyle changes can be challenging to maintain long-term, especially in children with no current disease.

We also found that respondents preferred minimally invasive tests, such as saliva, blood, and stool compared to more invasive procedures such as colonoscopy with biopsies and endoscopy. Respondents, rightfully so, appear to understand that more invasive procedures carry a greater risk for complications. These findings mirror preferences for less invasive tests among established patients with IBD.^35–37^ As research informs the development of diagnostic tests for IBD, it is critical that the tests be accessible, affordable, patient-friendly, and accurate.

### Limitations

There are a number of limitations to this study. The scenarios presented in the survey were hypothetical; tests to predict IBD and treatments to prevent IBD do not yet exist. The benefits and risks of any tests and treatments once developed will likely influence people at risk in ways we were unable to measure given hypothetical scenarios. Responses in this study may not accurately reflect choices that would be made in a real-life situation as the gap in intentions and behavior is well documented.^38–40^ Respondents were primarily patients with IBD and their choices may not reflect the choices of first-degree relatives who would be eligible for a predictive test or preventive treatment. The survey did not ask about smoking status among the individuals at risk of developing IBD. It is possible that many of the respondents who reported they would quit smoking to prevent IBD are already non-smokers. Given that an estimated 18% of adults with IBD are current smokers,^41^ future surveys should include questions about smoking status among those at risk of developing IBD. Specifically, it should ask current smokers about their willingness to quit smoking to prevent IBD. Focusing the analysis on current smokers will provide a valid description of how willing at-risk respondents are to this as an intervention.

As research informs the understanding of prevention of IBD, lifestyle and environmental interventions beyond diet and exercise may be developed to reduce risk of developing the disease, reduce severity, and delay onset. Novel pharmacological interventions beyond those proposed in this study, such as new targets for small molecules and biologics and novel drug delivery systems, such as implants, patches, and microneedles may be developed as prevention treatment options. Attitudes toward these novel treatments should be explored in future studies.

Although survey outreach was via several diverse patient advocacy organizations, most respondents heard about the survey from the lead organization (73%) and respondents reflect the population that typically engages with the lead organization (e.g., white, female, college educated). The responses may therefore not be reflective of a wider, more diverse, IBD community. Additionally, an online medium that requires English fluency may limit responses from those with health literacy, language or communication barriers. Future studies should aim to include a more diverse population to better understand differences in attitudes toward prediction and prevention among more diverse racial and ethnic groups. As tests to predict risk of developing IBD and prevention treatments are developed, the IBD field should employ best practices in communicating the benefits of testing and treatment learned from partnerships working to screen for disease and prevent disease progression in other disease states, such as type 1 diabetes, rheumatoid arthritis, and multiple sclerosis. To improve the comprehension of patients and families regarding the relationship between predictive tests and treatments aimed at reducing the risk of developing IBD and delaying onset and progression, it is essential to implement standardized phrasing, branding, and messaging across all communication channels, as exemplified by TrialNet.^42^ Communications must be developed in collaboration with patients and their families to ensure that strategies resonates and align with the values and preferences of patients, caregivers, and first-degree relatives.

## Conclusion

In conclusion, IBD screening and prevention are accepted by a large majority of respondents to this survey, and non-invasive screening and lifestyle interventions were the preferred modalities to achieve this. This provides support for funders, regulators and sponsors to develop a roadmap for future interception studies with patient/caregiver input.

## Supplementary Material

otag004_Supplementary_Data

## Data Availability

Data available upon request from the corresponding author.
