# Correction to: Patients’ attitudes to disease prevention in inflammatory bowel disease: a US-based survey

**DOI:** 10.1093/crocol/otag043

**Published:** 2026-05-20

This is a correction to: Mary Harkins-Schwarz, Catarina Bravo, Manasi Agrawal, Jean Frederic Colombel, Ryan Ungaro, Laura Wingate, Joana Torres, Alan Moss, Patients’ attitudes to disease prevention in inflammatory bowel disease: a US-based survey, Crohn’s & Colitis 360, Volume 8, Issue 1, January 2026, otag004, https://doi.org/10.1093/crocol/otag004

The third co-author’s name has been corrected to read: “Manasi Agrawal”.

