# Supplementary material for: Patients’ attitudes to disease prevention in inflammatory bowel disease: a US-based survey
Source: Crohns Colitis 360. 2026 Feb 3;8(1):otag004. doi: 10.1093/crocol/otag004 (PMC12865844; doi:10.1093/crocol/otag004)
Supplement: otag004_Supplementary_Data [file otag004_supplementary_data.zip › US.PreventionSurvey.Final.pdf]

Introduction

The Crohn’s & Colitis Foundation -- in partnership with Girls with Guts, Color of Gastrointestinal Illness (COGI), ImproveCareNow (ICN), Crohn’s and Colitis Young Adult Network (CCYAN) and South Asian IBD Alliance (SAIA)-- is conducting a survey to gather opinions and attitudes from individuals with IBD and their families related to the concept of preventing Inflammatory Bowel Disease. The results of the survey will aid the Foundation and the partnering organizations in understanding preferences related to potential tests, diagnostics and treatments. The findings will be used to inform research and advocacy over the next several years.

The survey is open to:

- **Adults at risk of developing IBD because they have a first degree relative (parent, sibling, child) with IBD,**
- **Adults who have a child who is at risk of developing IBD because the child has a first degree relative (parent, sibling) with IBD, and**
- **Adults who have IBD (or whose partner has IBD)**

Completing the survey is voluntary. Your responses to this survey will be anonymous. The findings from this study will be reported in summary form so that the participants cannot be identified. We encourage you to respond candidly.

There is no guarantee of a direct benefit to you from completing the survey. However, the information you provide may help inform research and advocacy related to risks for and prevention of IBD.

The survey will take approximately 10 minutes to complete.

Thank you in advance for taking the survey. Your answers can make a real difference in the lives of people at risk of developing inflammatory bowel disease.

Please feel free to contact us for answers to specific questions.

About you

Before we start the survey, we would like to know some information about you.

* 1. Select the situation that best applies to you:

- ◯ I have inflammatory bowel disease (IBD)
- ◯ I do NOT have IBD, but have a child with IBD
- ◯ I do NOT have IBD, but I have a first-degree relative (mother, father, sister, or brother) diagnosed with IBD
- ◯ I do NOT have IBD, but have a partner/spouse diagnosed with IBD
- ◯ None of the above

* 2. Are you 18 years or older?

- ◯ Yes
- ◯ No

The next set of questions are about the person who has IBD. That may be you, your partner, your child, or another first degree relative. In this survey, we will call the person with IBD the "affected person". If you have multiple family members with IBD, please answer the questions thinking about the affected person with the most severe disease.

3. At what age was the affected person diagnosed with IBD?

Please enter a number. For example, if you, your child, or family member were diagnosed at 17 years old, please enter "17".

4. What type of IBD does the affected person have?

- Crohn’s Disease
- Ulcerative Colitis
- Indeterminate Colitis (IBD Unclassified)
- I don’t know

5. Has the affected person ever had or is currently undergoing treatment for IBD?

- Yes
- No
- Unknown/Not sure

Treatments

6. Which of the following type(s) of treatment has the affected person received? Check all that apply.

- ☐ Aminosalicylates (5-ASAs) – oral, suppositories, enemas (eg Apriso, Asacol, Canasa, Lialda, Mesalamine, Pentasa, Rowasa)
- ☐ Corticosteroids – oral, suppositories (eg, prednisolone, Entocort)
- ☐ Immunomodulators (e.g., Azathioprine, Imuran, Mercaptopurin)
- ☐ Antibiotics – (e.g., Cipro, Flagyl)
- ☐ Biologics/Biosimilars – Infliximab, Adalimumab, Vedolizumab, Ustekinumab (eg Remicade, Inflectra, Humira, Entyvio, Stelara)
- ☐ Targeted Synthetic Small molecule (Etrasimod, Tofacitinib, Oxanimod, Upadacitinib)
- ☐ Other
- ☐ Unknown/Not sure

Impact of IBD

7. Has the affected person ever been hospitalized for IBD-related reason(s)?

- ◯ Yes
- ◯ No
- ◯ Unknown/Not sure

8. Has the affected person ever had any surgery for IBD-related reason(s)?

- ◯ Yes
- ◯ No
- ◯ Unknown/Not sure

9. What do you consider to be the impact that IBD has on the affected person's life?

- ◯ Very significant, interferes with simple day-to-day activities
- ◯ Significant, interferes with some work (work/school), social life or recreational activities
- ◯ Little, rarely interferes with any activity
- ◯ None, child/family member or I have a normal life without any limitations

About IBD

The next set of questions ask about your thoughts and opinions about IBD.

10. What is your opinion on the following statement: “Even if a patient with IBD follows the doctor's instructions and adheres to medication, they will never have a normal quality of life, that is, they will never be able to work or travel without restrictions.”

- ◯ I totally agree
- ◯ I agree
- ◯ I have no opinion
- ◯ I disagree
- ◯ I totally disagree

11. IBD is a disease caused by: (check all that apply)

- ☐ Infectious disease (something someone can catch, like the flu)
- ☐ Immune-mediated or autoimmune disease (body's immune system mistakenly attacks its own cells, tissues, or organs)
- ☐ Genetics (disease is inherited or passed down from one or both parents to their offspring)
- ☐ Unhealthy diet
- ☐ Unhealthy lifestyle
- ☐ Stress
- ☐ Drugs (antibiotics and medications)
- ☐ External/environmental factors
- ☐ Cancer
- ☐ Don’t know/Prefer not to answer

12. The cause of IBD:

- ◯ Is already well-known and studied
- ◯ Is still not well known despite all the research
- ◯ Don't know

13. IBD:

- ◯ Is a genetic disease with a well-known transmission from parents to children
- ◯ Is a disease without a known genetic transmission, but with increased risk in direct relatives (1st degree relatives, such as mother, father, sister, brother) of affected patients
- ◯ There is no increased risk of IBD in relatives of affected patients
- ◯ Don't know

14. The risk of a 1st degree (mother, father, sister, or brother) of a patient with IBD developing the disease is:

- ◯ Same as the general population
- ◯ Lower than the general population
- ◯ About 3 to 4 times higher than the general population
- ◯ Don’t know/prefer not to answer

15. It is currently recognized that IBD has a pre-clinical phase (no symptoms but inflammation has started in the bowel), which begins at least a few years before symptom onset. What could be the benefit(s) of clinical research to understand this pre-clinical phase? Check all that apply.

- ☐ Improve knowledge of IBD in general
- ☐ Lead to the development of new therapies
- ☐ Disease prediction (predict the risk of the disease developing)
- ☐ Prevention of disease (try to prevent the disease from developing)
- ☐ Earlier diagnosis which may prevent severe disease and complications

16. Which statement do you agree with:

- ◯ Current medications can cure IBD
- ◯ Medications can control symptoms and inflammation, but not cure IBD

17. Is there currently any medication, supplement, or diet capable of preventing the disease from developing in a healthy individual?

- ◯ Yes, there are preventive treatments (medications, diet, or other treatments)
- ◯ No, there is no way to prevent IBD

Tests to predict risk

The next questions ask about how you feel about tests that may predict the risk of developing IBD and treatments and lifestyle changes that may prevent or reduce the risk of developing IBD. If you have been diagnosed with IBD, please answering these questions thinking about your child or another close family member who has not been diagnosed with IBD.

18. If there were any tests that could predict your or your family's risk of developing IBD in the next few years, would you like to take them?

- ◯ Yes, regardless of test accuracy
- ◯ Yes, but it would depend on the accuracy of the test
- ◯ No, I would rather not know
- ◯ I am not sure

The next questions ask your thoughts about preventive tests. Below are brief descriptions of each type of test.

- A saliva test is a way to check for disease or condition by analyzing a small sample of your spit.
- A blood test is a way to check for disease or condition by analyzing a small sample of your blood.
- A stool test is used to check for disease or condition by analyzing a small sample of your poop.
- A CT is a special type of X-ray that takes detailed pictures of the inside of your body.
- An MRI is a machine that takes detailed images of your body using magnets and radio waves. Patients lie on a table and slide into the machine for the images to be taken
- Intestinal ultrasound - A doctor or nurse uses a small device and rubs it on your belly. The test using sound waves to create images of a your intestine. It does not use radiation.
- A colonoscopy with biopsy is a procedure during which a healthcare professional uses a flexible tube with a camera to look inside your colon and rectum. Your healthcare professional may take small tissue samples if needed to check for disease.
- An endoscopy is a procedure during which a healthcare professional uses a flexible tube with a camera to look inside your digestive tract. During an upper endocsopy your provider may examine your esophagus, stomach, and beginning of your small intestine. During a flexible sigmoidoscopy your provider may examine your the lower part of your large intestine, including your rectum and sigmoid colon.
- A pill cam is a small, device that you swallow. It takes pictures of your digestive tract.

19. Given the different degrees of invasiveness, risks and other factors, please rank the following test(s) to predict the risk of developing IBD in order of how willing you or your family would be to take them. (1 being the test you/your family member is most willing to take and 9 being the test you/your family member is least willing to take.)

☐ Saliva test

☐ Blood test

☐ Stool test

☐ Computed tomography (CT)

☐ Magnetic resonance imaging (MRI)

☐ Intestinal ultrasound

☐ Colonoscopy with biopsies

☐ Endoscopy

☐ Pill cam

Advantages and disadvantages of tests

20. What do you think would be the advantages of taking a test to predict if you or your family member will develop IBD? Check all that apply.

- ☐ Possibility of changing lifestyle (e.g., diet, exercise, meditation), taking medication, or having surgery to try to reduce the risk that I or my family member will develop IBD
- ☐ Possibility to explain to my family member in a timely manner or to learn more about what IBD is and what it is like to live with the disease
- ☐ Possibility of doing some activities or trips before the disease appears, which would be more difficult to do with the disease
- ☐ Possibility for me or my family member to start testing so that the disease is diagnosed, and treatment is started as early as possible
- ☐ None

21. What do you think would be the disadvantages in carrying out a test to predict if you or your family member will develop IBD? Check all that apply.

- ☐ The test may not give me 100% correct results
- ☐ The test can generate anxiety because with a high risk I would always be waiting for the disease to appear in me/my family member at any time
- ☐ Worry about the risk that I/my family member will develop the disease when there is still no way to act and prevent the disease
- ☐ None

Preventive treatments

The next questions are about preventive strategies or treatments. Preventive strategies or treatments are things that might be able to reduce the risk of you or your child developing IBD. They may include things like changing your diet, exercising, or taking a medication. If you have been diagnosed with IBD, please answer these questions thinking about your child or another close family member who has not been diagnosed with IBD.

22. If there were some kind of preventive treatment capable of reducing the risk of you or your family member developing the disease, would you like to do it?

- ○ Yes
- ○ No
- ○ It would depend on the effectiveness, difficulty, and risks of the treatment/intervention.

23. What should be the goal of a preventive treatment for you or your family member to accept doing it? Check all that apply.

- ☐ Reduce or eliminate the risk of the disease developing
- ☐ Delay the development of the disease
- ☐ Make the disease less severe

24. Please rank the following preventive treatments in order of how willing you or your family member would be to take them. (1 being the treatment you/your family member is most willing to take and 9 being the treatment you/your family member is least willing to take.)

- ☐ Quit smoking
- ☐ Physical exercise program
- ☐ Diet based
- ☐ Probiotics
- ☐ Dietary supplements
- ☐ Oral antibiotics
- ☐ Stool transplantation
- ☐ Immunosuppressive/immunomodulatory drugs in oral tablet format
- ☐ Immunosuppressive/immunomodulatory drugs in the form of subcutaneous/intravenous injections

25. How effective would the preventive treatment/ strategy have to be for you or your family member to accept it?

- ◯ 100% risk reduction (completely gets rid of the risk of developing the disease)
- ◯ 80% risk reduction (8 out of 10 people stop developing the disease)
- ◯ Risk reduction 50% (5 out of 10 people stop developing the disease)
- ◯ It depends on the type of treatment/intervention
- ◯ Even if the risk of developing the disease remained the same, decreasing the impact of the disease or delaying its development would already be worth it

Side effects

26. If you or your family member were to use a treatment to prevent IBD from occurring, at what level would you consider the risk of **minor adverse effects** to be acceptable? Examples of mild adverse effects: symptoms such as headache, nausea or vomiting, diarrhea, tiredness, abdominal pain that resolve spontaneously.

- ◯ Fewer than 1 in 10 people experience minor adverse effects from the preventive treatment
- ◯ Up to 1 in 10 people experience minor adverse effects from the preventative treatment
- ◯ Up to 2 in 10 people experience minor adverse effects from the preventative treatment
- ◯ Up to 4 in 10 people experience minor adverse effects from the preventative treatment.
- ◯ Would accept a higher risk (out of 10 people, 6 or more experience minor adverse effects) if the treatment/intervention were 100% effective in preventing the disease

27. If you or your family member were to use a treatment to prevent IBD from occurring, at what level would you consider the risk of **serious adverse effects** to be acceptable? Example of serious adverse effects: complications requiring hospitalization, invasive tests or intravenous treatment

- ☐ Up to 1 in 100 people will experience serious adverse effects from treatment to prevent IBD from occurring
- ☐ Up to 5 in 100 people will experience serious adverse effects from treatment to prevent IBD from occurring
- ☐ Up to 10 in 100 people will experience serious adverse effects from treatment to prevent IBD from occurring
- ☐ Would accept a higher risk (out of 100 people, 11 or more will experience serious adverse effects) if the treatment/intervention were 100% effective in preventing the disease

28. What duration of a preventive **pharmacological treatment** (medicine) would you consider reasonable for you or your family member to accept it?

- ◯ 8 weeks or less
- ◯ 9 weeks - 6 months
- ◯ 7 months - to 1 year
- ◯ More than 1 year – 4 years
- ◯ 5-10 years
- ◯ Would accept a treatment for life, if it was 100% effective in preventing the disease

29. What duration of a preventive **non-pharmacological intervention** (physical exercise, diet, quitting smoking, food supplements) would you consider reasonable for you or your family member to accept it?

- ◯ 8 weeks or less
- ◯ 9 weeks - 6 months
- ◯ 7 months - to 1 year
- ◯ More than 1 year – 4 years
- ◯ 5-10 years
- ◯ Would accept an intervention for life if it was 100% effective in preventing the disease

The next question asks about your thoughts about the risk of developing IBD. **If you have IBD, please answer thinking about your child or another close family member who has not been diagnosed with IBD.**

30. On a scale of 1 to 10, how likely do you think it is that you or your family member will develop IBD in your lifetime?

1 Not at all likely — 10 Extremely likely

31. Please use this space to share your thoughts about predictive tests and preventive treatments/intervention that has not been addressed in the previous questions.

About your background

Now a few questions about your background. Answers to these final questions will be used to describe the type of people completing this survey.

32. Indicate how you first heard about this survey.

- ◯ Girls with Guts
- ◯ Color of Gastrointestinal Illness
- ◯ ImproveCareNow
- ◯ Crohn’s & Colitis Foundation
- ◯ Crohn’s And Colitis Young Adult Network
- ◯ South Asian IBD Alliance
- ◯ My physician/my first-degree relative’s physician
- ◯ My family member affected with IBD.
- ◯ Other

33. Which of the following describes you and your family? (Check all that apply)

- ☐ I have IBD
- ☐ My father has IBD
- ☐ My mother has IBD
- ☐ My brother/sister has IBD
- ☐ My partner/spouse has IBD
- ☐ I have a child diagnosed with IBD
- ☐ I have a child who does NOT have IBD
- ☐ I or my partner is pregnant or we are planning on having children

34. How many of your first-degree relatives (mother, father, sibling, child) have been diagnosed with IBD? *Do not include yourself.*

____

35. Select your age (years):

____

36. Select your gender:

- ◯ Male
- ◯ Female
- ◯ Non-binary
- ◯ Other
- ◯ Prefer not to answer

37. Select your state of residency

[ ]

38. Select your race. Check all that apply.

- ☐ White
- ☐ Black or African American
- ☐ American Indian or Alaska Native
- ☐ Asian
- ☐ Middle Eastern or Northern African
- ☐ Native Hawaiian or Other Pacific Islander

39. Select your ethnicity:

- ○ Hispanic
- ○ Non-Hispanic or Latino

40. What was the highest level of education you completed?

- ○ Some school but no high school diploma
- ○ High school diploma or GED
- ○ Some college credit but no degree
- ○ Associate degree (such as AA, AS)
- ○ Bachelor’s degree (such as BA, BS)
- ○ Master’s degree or higher
- ○ Other
- ○ Prefer not to disclose

Thank you

Thank you for taking the time to complete this survey. The findings from this survey will be used to aid the Foundation and partnering organizations – Girls with Guts, Color of Gastrointestinal Illness (COGI), ImproveCareNow (ICN), Crohn's and Colitis Young Adult Network (CCYAN) and South Asian IBD Alliance (SAIA) – in understanding preferences related to potential tests, diagnostics and treatments. The findings will be used to inform research and advocacy programs over the next several years.

Please share this survey link with your family or friends who:

- **Do not have IBD, but have a first degree relative (parent, sibling, child) with IBD**
- **Have a child who is at risk of developing IBD because the child has a first degree relative (parent, sibling) with IBD**
- **Have IBD (or whose partner has IBD)**
