# Supplementary figures and images for: Patients’ attitudes to disease prevention in inflammatory bowel disease: a US-based survey
Source: Crohns Colitis 360. 2026 Feb 3;8(1):otag004. doi: 10.1093/crocol/otag004 (PMC12865844; doi:10.1093/crocol/otag004)

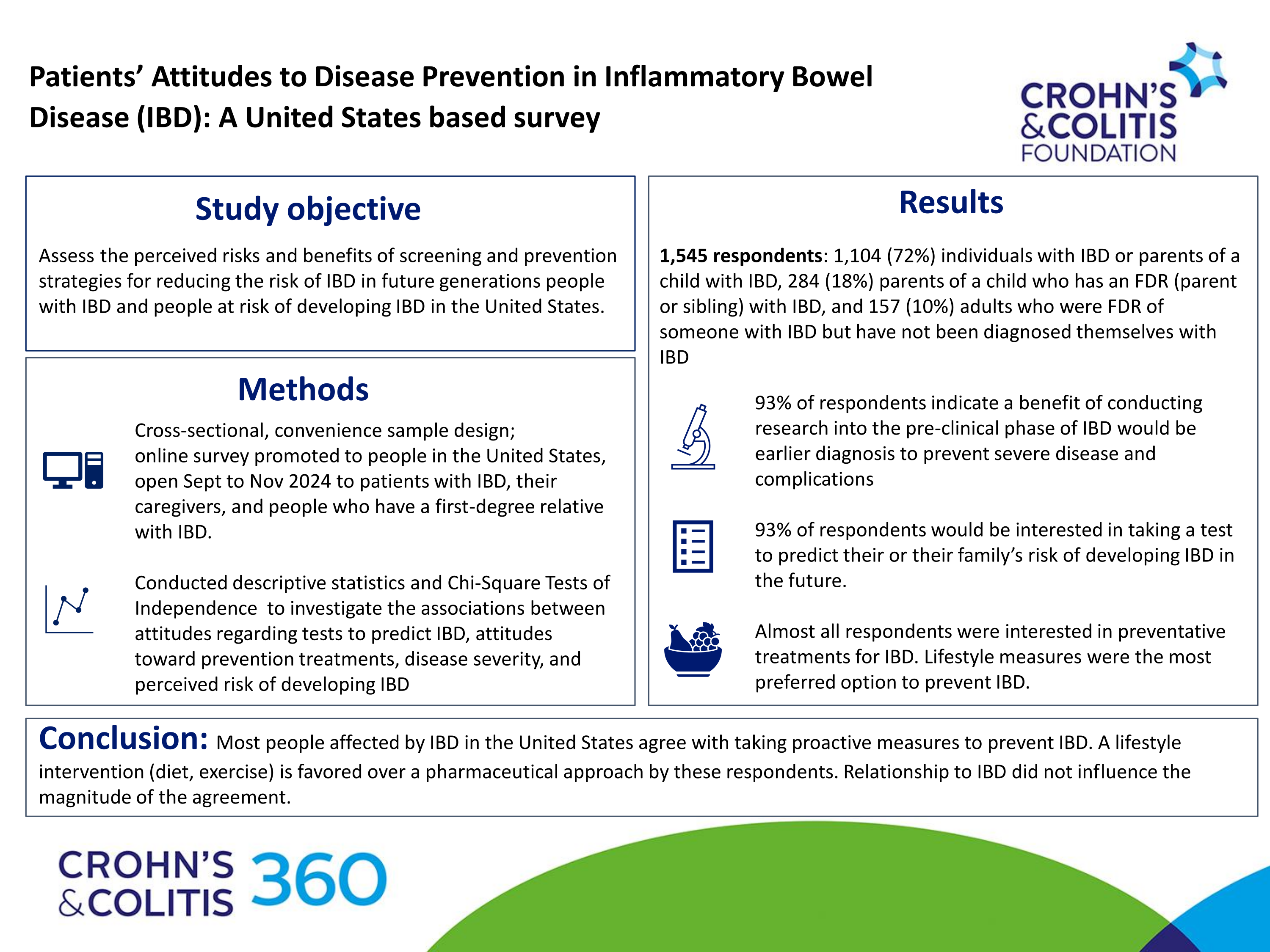

Supplement: otag004_Supplementary_Data [file otag004_supplementary_data.zip › CC360 Visual Abstract (1).tif]
